# Trunk picking from a truncating menu: Dry season forage selection by Asian elephant in a multi-use landscape

**DOI:** 10.1371/journal.pone.0271052

**Published:** 2022-07-08

**Authors:** Priyanka Das, Aritra Kshettry, H. N. Kumara

**Affiliations:** 1 Sálim Ali Centre for Ornithology and Natural History, Coimbatore, Tamil Nadu, India; 2 INSPIRE-Fellow, Department of Science and Technology, Government of India, New Delhi, India; Centre for Cellular and Molecular Biology, INDIA

## Abstract

Elephants show a strong selection towards areas with high foraging opportunities at the landscape level making top-down decisions by first selecting patch types within landscapes and finally species within them. Understanding forage selection in a multi-use landscape is critical for prioritising patches for habitat management, ensuring availability of selected forage, helping in minimizing pressure on food crops and subsequent negative interactions with people. We assessed dry season forage selection in a multi-use landscape of West Bengal state, India. Relative forage use and relative plant species availability ratio were calculated to assess forage selection in a multi-use landscape comprising of the forest, tea estates, agricultural land, and human settlement. Forage use was assessed using the opportunistic feeding trail observation method (150.01 km). Stratified random sampling was used to assess plant species availability using the quadrat method (123 plots of 0.1 ha each). Among 286 plant species recorded, 132 plant species were consumed by elephants. A majority (80.21%) of plant species were consumed more than the proportional availability thereby showing selective foraging during the dry season in the study area. From forest to semi-open forest and open forest, canopy layer tree density and the total number of species decreased whereas invasive species density increased. This indicates the high impact on the forage species availability for elephants and the requirement of appropriate habitat management strategies. The presence of 32.14% of the selected forage species in human-use landscape alone demands the development of conservation interventions. This is the first study to assess forage selection by elephants in a multi-use landscape and used to prioritise conservation and management strategies at a landscape level.

## Introduction

The Asian elephant is endangered with its historical widespread distribution range being shrunk to small pockets in 13 countries in south and south-east Asia due to habitat loss, fragmentation, poaching, being taken into captivity, and negative interactions with humans [1–4]. Being a wide-ranging species with vast space and resource requirements, elephants depend on vast stretches of land rather than a few forest patches [2, 3]. However, globally only 51% of their present range is covered by forests, and 16% fall under the purview of legal protection [3]. Hence, they are increasingly found in land-use mosaics leading to competition over resources, economic losses, and even human and elephant casualties in extreme cases [5–7]. This makes it imperative to focus conservation efforts for Asian elephants at a landscape level which provides scope for a cost-effective way to achieve a significant set of conservation goals in a timely fashion. However, conservation investment and planning in lands outside the Protected Area needs to be based on empirical evidence and prioritization [8, 9]. One such way could be through quantifying resource selection in a multi-use landscape as availability and distribution of resources seem to primarily influence the occurrence and movement of elephants in a particular landscape and could explain associated negative interactions with people [2, 10].

Forage could be the most important resource in this regard as elephants spend 70–90% of their time foraging and consume around 150 kg of forage daily [2]. Furthermore, on a landscape scale, elephants are known to make top-down foraging decisions where they first select landscapes followed by patch types with high foraging opportunities and then plant species present [11, 12]. Local densities of elephants are known to correlate with the fruiting season of specific plants as well [13–15]. On the other hand, crop foraging by elephants is the primary driver of negative interactions with humans with estimated damage of 0.8–1 million hectares of crops every year in India in human-dominated areas [6, 16].

Although forage use by Asian elephant is well documented [17–25], very less is known about forage selection [26, 27]. Resource use is defined as the quantity of the resource that is utilized by an animal (or population of animal) in a fixed period of time and resource availability is the quantity accessible to the animal (or the population of an animal) during that same period [28]. Selection is defined to be strictly a binary decision with outcomes of use or non-use of a resource unit [29]. As the availability of plants in a landscape determines foraging decisions by elephants [2], it is important to base inferences on the ecological role, needs, and related conservation intervention on forage selected and not merely forage used. Moreover, knowledge on forage use is mostly restricted to savannah habitats and tropical dry forests while very less is known about tropical moist forests and land-use mosaics, despite the fact that forage use by elephants greatly varies across habitat types [25].

Understanding forage selection will enable us in ensuring the availability of selected forage species by effective conservation and management of remnant forest patches in multi-use landscapes. It will also help in devising appropriate measures to prevent negative interaction with people when elephants go to forage in human-dominated areas. Assessment of forage selection would also be the first step towards understanding various other ecological aspects about an elephant population such as i) carrying capacity of a landscape as the availability of food resources is a major determinant [15], ii) role in seed dispersal which may ascertain forest species composition [30, 31], and iii) if a particular population is undergoing nutritional stress [32].

We studied dry season forage selection by Asian elephants in a multi-use landscape with the objectives of assessing forage use and availability and quantifying selection in different land-use types. Northern districts of West Bengal in India were deemed ideal for this study as the landscape has fragmented forests in a mosaic of tea plantations, agricultural land, human settlements, [33] and experiences one of the highest negative interactions between humans and elephants in Asia [7]. This site provides an additional opportunity to address the paucity of knowledge on foraging decisions in the moist tropical forest.

## Study area

**Fig 1:**
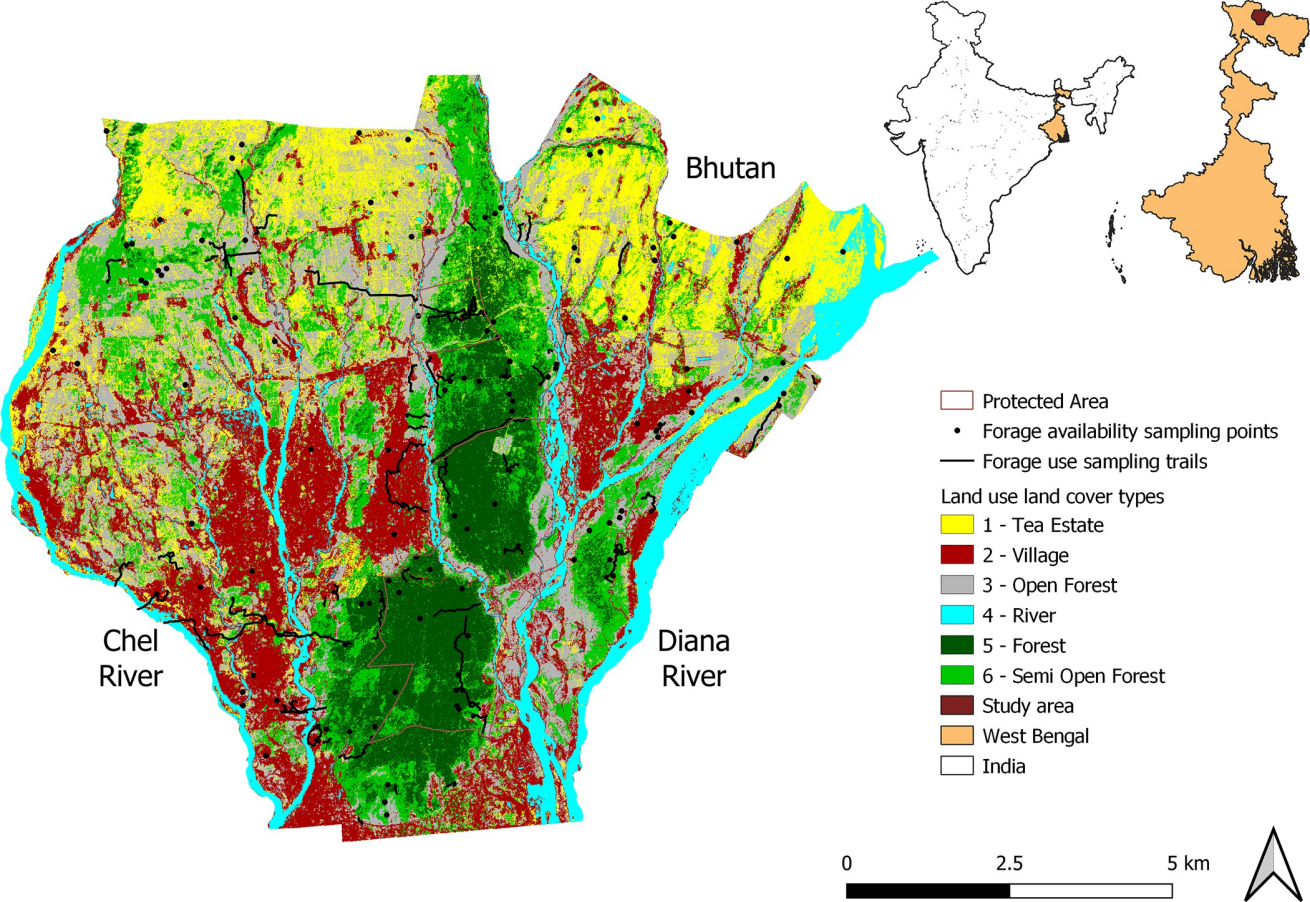
Land use land cover classification, forage use sampling trails, forage availability sampling points in the study area of West Bengal State.

The study site lies in West Bengal, India, bordered by Chel river in the west, Diana River in the east, and Bhutan in the north, and comprises an area of 908.81 km^2^ (Fig 1). It is a multi-use landscape including the Protected Areas of Chapramari Wildlife Sanctuary and Gorumara National Park, reserved forests of Jalpaiguri Forest Division and Kalimpong Forest Division, tea estates, agricultural land, and human settlements of Jalpaiguri District (Fig 1). A multitude of reasons led to the fragmentation of forest in the landscape like the clearing of natural forest for timber plantations and tea estates by the erstwhile colonial administration, settlement of Taungya cultivators in the 19th century, establishment of army units in the 1960s, and anthropogenic pressure from the growing human population [22, 33].

The region is part of the East Himalayan biodiversity hotspot [34] with a rich faunal and floral diversity [33]. The major forest types are northern tropical semi-evergreen forest and tropical moist deciduous forest and the grassland type is east Indian alluvial grassland [35]. During the study period, the grasslands were burnt and cleared as part of the regular habitat management program of the Forest Department to prepare them for the new plantations. Apart from natural forest, there are monoculture and mixed plantations which mostly comprise *Tectona grandis*, *Shorea robusta*, *Lagerstroemia speciosa*, *Ailanthus integrifolia*, and *Acacia catechu*.

The annual temperature ranges between 7.8⁰C-37.9⁰C (http://jalpaiguri.gov.in/html/disprof.html, accessed July 2019) and the annual rainfall is 3160 mm [33]. The region has a high human population density of 701 people/ km2 (http://jalpaiguri.gov.in/html/census.html,census2011.co.in/census/district/1-darjiling.html, accessed August 2019). The main crops grown in the landscape are paddy, maize, and jute. However, during the study, the agricultural fields were mostly uncultivated with few households growing *Brassica nigra*, *Areca catechu*, *Musa sp*. for consumption.

The elephant population in the region is the western most extension of the north-eastern Indian elephant population that ranges from the Sankosh river at Assam-West Bengal border to Mechi River at Indo-Nepal border [22] and comprises ~488 individuals spread across a forest area of 1933 km^2^ [36]. Around 57% of the landscape used by elephants remains outside the Protected Areas thereby underscoring the importance of non-protected areas for movement and connectivity of this sub-population [7]. The landscape experiences very high negative interactions between humans and elephants with an estimated number of humans killed and injured annually by elephants being 47 and 164 respectively while average annual crop damage by elephants summed up to 2078 hectares per year between 2006–2016 [37].

## Methods

### Study design

For assessing forage selection, Manly’s type I study design was used where measurements are made at the population level with used-versus-available resource unit protocol [28]. As assessment of resource use and resource availability should be independent of each other [28], forage use was assessed using the feeding trail observation method in different land-use types [26, 27] and plant species availability was assessed using the quadrat method by generating random points in different land-use types [38]. Forage selection was assessed by calculating the ratio of relative forage use and relative plant species availability [26].

### Land use and land cover classification

To perform land use and land cover (LULC) classification, Sentinel 2 imagery of the study site was procured from the United States Geological Survey (https://earthexplorer.usgs.gov/). The images were from the dry season of 2019 with less than 10% cloud cover. Image processing and stitching were done using ERDAS IMAGINE 2014 and image clipping was done using QGIS version 3.6.0. The study area was classified into six LULC types which were forest, semi-open forest, open forest, tea estate, village, and river (Fig 1). Forest represented areas with a closed canopy, dense understory, and moist floor with thick leaf litter. Semi-open forest represented areas with partially open canopy, where the understory has disappeared and mostly constituted of plantations. The open forest had a completely open canopy with sparsely spaced trees. Forest, semi-open forest and open forest were protected land (Reserved Forest or National Park or Sanctuary) while village and tea estate were private land or revenue land. Thirty training sites were used for each LULC type to perform ground-truthing (S1 Table). The supervised classification approach (Spectral Angular Mapper) was used to classify the LULC types with Semi-Automatic Classification Plugin (SCP) in QGIS version 3.6.0. The accuracy assessment that was performed in SCP showed 61.40% accuracy and the kappa hat classification was 0.53 which indicates moderate agreement.

### Field method and data analysis

#### Plant species identification

Plant species were identified by their vernacular names (Nepali, Bengali, and Sadri) by the field associate and other Forest Department personnel who accompanied the researcher. Scientific names of the plant species were then found with the help of existing literature [22, 39–41] and online portals like ‘Flowers of India’ (http://www.flowersofindia.net/) and ‘Indian Biodiversity Portal’ (https://indiabiodiversity.org/). In addition, plant species photographs were taken, which were later identified by botanists. The latest accepted scientific names were used from the online portal ‘The Plant List’ (http://www.theplantlist.org/). All the species of the genus Musa were clubbed and have been written as *Musa sp*.

#### Forage use

The feeding trail observation method [26, 27] was used to collect forage use data as direct observation was not possible due to dense vegetation and poor visibility. Additionally, elephants are known to improve their foraging efficiency by following trails created by repeated movements to and from dependable resources [42]. The feeding trails were located based on information about elephant presence received from the information network that was built with Forest Department, tea estate workers, and villagers. These trails were visited within two days of use by the elephants, so that the fresh feeding signs could be observed. Indirect signs like footprints, dung piles, and body rubbing marks on trees were used to follow the feeding trail and the route was recorded using handheld global position system unit (Garmin eTrex 30x). The trail length sampled varied because of the absence of indirect signs after a certain distance, or the presence of elephants on the trail while walking on it, or due to forest jurisdiction issues. Plants showing signs of elephant feeding on either side of the trail were recorded. Indirect evidence of plants consumed by elephants comprised chewed vegetation, debarked and broken twigs and branches, scratched posts, foot and body marks on soil [20], uprooted plants, scattered leaves with no stem and for grass, clumps of their roots were found with the blades fed on. Along with the plant species consumed, the following things were recorded: i) variety of the plant consumed (climber, herb, shrub, tree, bamboo, palm, and orchid), ii) part of the plant consumed (leaf, stem, branch, bark, and root) (S2 Table) as elephants are sometimes selective of the plant part consumed [43], iii) the LULC type of the trail, iv) length of the trail using GPS, v) information on whether the trail was used by a herd or male. Male includes solitary male elephants or multi-male group of elephants henceforth referred to as male and herd include a matriarchal group with adult females, subadults, infants and juveniles of both sexes and henceforth referred to as a herd. The information on whether the trail was used by a male or herd was obtained from the informer and verified on the field. If there were multiple footprints of different sizes, we assumed a feeding trail to be used by a herd and if there was a single footprint or footprint of only adult elephants, we assumed a feeding trail to be used by a male/bull group.

Every time a plant species was consumed, it was assigned a value of one and at the end, the total number of times a plant species was consumed was summed up. The summed up values represented the total number of feeding signs for a plant species (S2 Table). The total number of feeding signs recorded in each trail was divided by the length of the trail (in km) for all the LULC types and this was termed as feeding frequency. Kolmogorov-Smirnov’s test was used to check the normality of the data and the Analysis of Variance test or Kruskal-Wallis test was used accordingly to check if the means are statistically indistinguishable wherever required [44]. Chi-square test for proportions [44] was used to check if the trail length used for males and herds in different land use and land cover types were statistically indistinguishable.

#### Plant species availability

Plant species availability was assessed using the quadrat method [38]. Random points were generated in the classified LULC types (forest, semi-open forest, open forest, tea estate, village) using QGIS version 3.6.0. Since misclassifications occurred between tea estate and open forest, polygons were made for the open forest in Google Earth Pro based on field knowledge before generating random points. Treating the random point as centre, a circular plot of 0.1 ha area was made to assess the canopy layer which included trees more than 30 cm girth, bamboos, palms, and bananas. Within this bigger plot, four 5 x 5 m subplots were made to assess the understory which included shrubs, tree saplings (less than 30 cm girth and more than 1 m height) and both soft stemmed and woody climbers; and five 1 x 1 m sub-plots were made to assess ground flora which included herbs, sapling of less than 1 m height and climbers on the ground. The abundance of all the species present within each plot was recorded.

The abundance of all the plant species was assessed for the entire sampled area. The abundance of understory and ground layer was later extrapolated for the 0.1 ha plot (S1 Text). The canopy layer tree density and invasive species density was calculated only in the forest, semi-open forest, and open forest which falls under the jurisdiction of forest department. Tea estate and village were not included in this analysis because these LULC types are already subjected to intensive management for maximizing production and there is very less scope for habitat management. The three most dominant invasive species (*Chromolaena odorata*, *Lantana camara*, and *Mikania micrantha*) were considered for the analysis. The density of canopy layer trees and invasive species was calculated by dividing the total abundance of canopy trees and invasive species available in each plot by the area of one plot (0.1 ha) for the forest, semi-open forest, and open forest.

#### Assessing the adequacy of sampling

**Fig 2:**
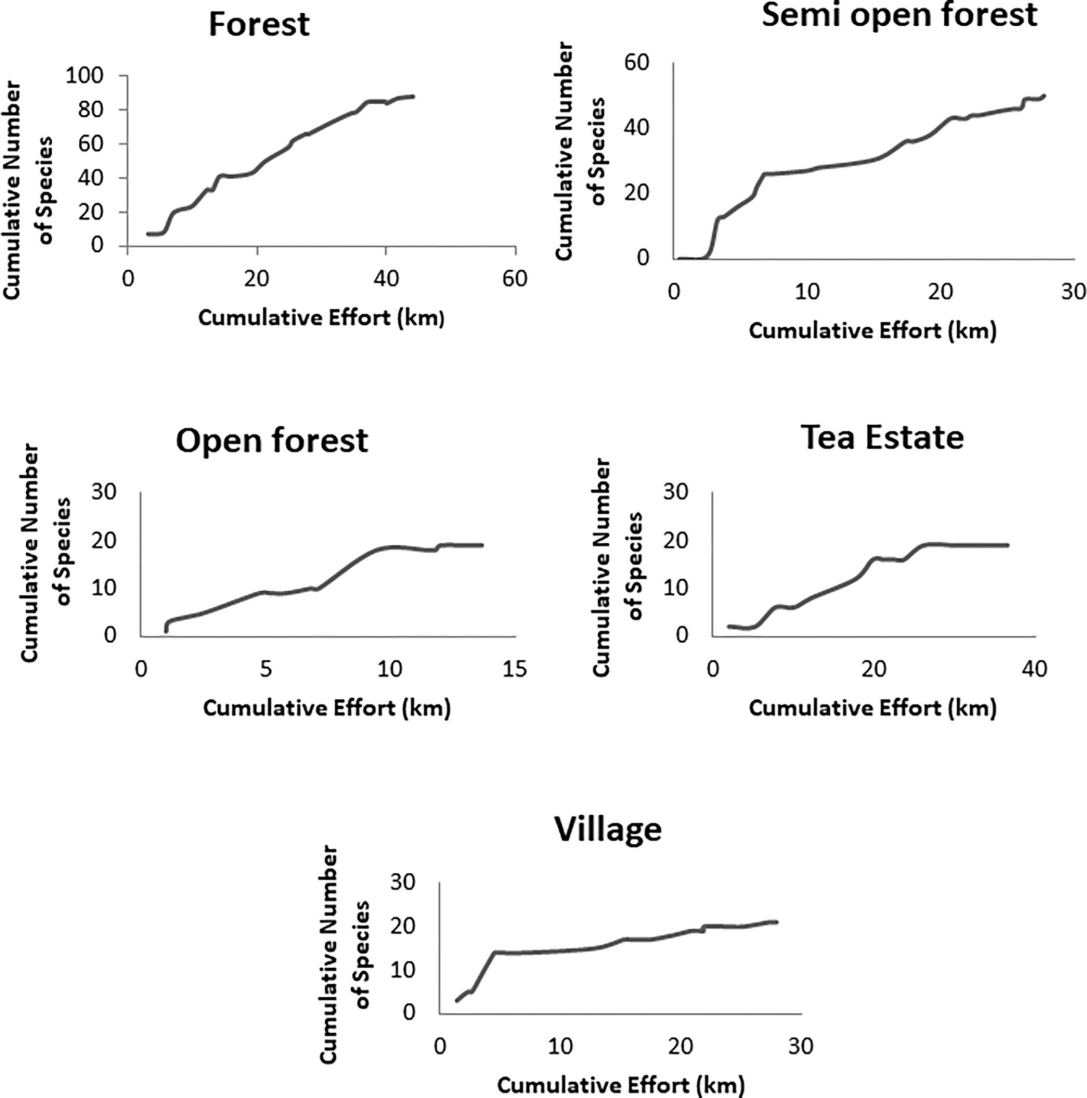
Species accumulation curve for forage use by Asian elephant in forest, semi-open forest, open forest, tea estate, and village.

**Fig 3:**
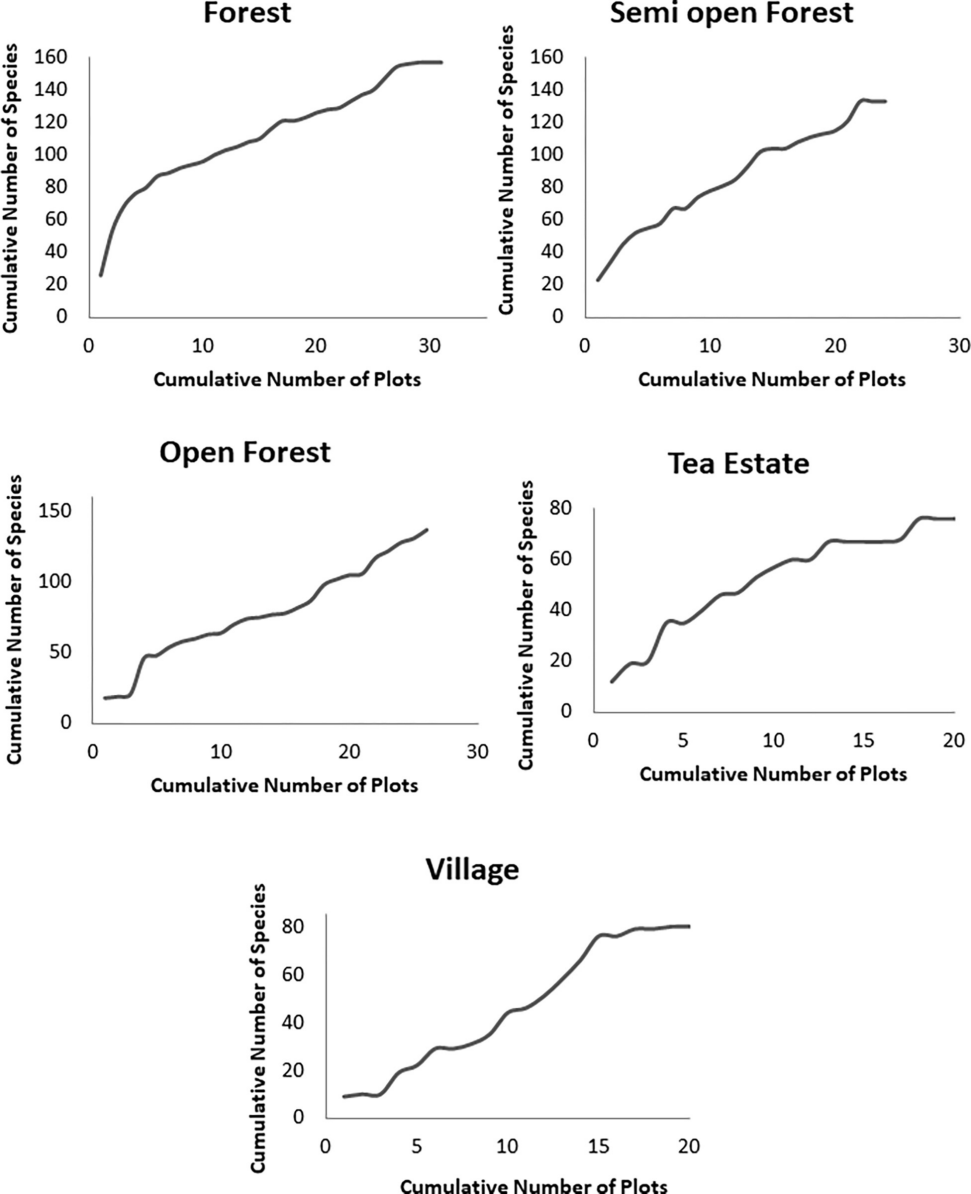
Species accumulation curve for plant species available in the forest, semi-open forest, open forest, tea estate, and village.

To assess the adequacy of sampling for forage use, species accumulation curves were plotted with the cumulative number of plant species that were recorded to be consumed against cumulative effort (in km) (Fig 2). To assess the adequacy of sampling for plant species availability, species accumulation curves were plotted with a cumulative number of plant species recorded against the cumulative number of plots sampled (Fig 3).

#### Forage selection

Forage selection was calculated using the relative availability (R_A_) of each species compared with their relative use (R_U_) [26].


$$R_{A}=N_{a}/T_{a},\;R_{U}=N_{u}/T_{u},\;Selection\;ratio=R_{U}/R_{A}$$

Where N_a_ is the number of available plants of a given species and T_a_ is the number of available plants across all species. N_u_ is the number of times a species was used and T_u_ is the total number of plants used for feeding across all species. Selection ratio > one indicates that the forage was utilised proportionately more than its availability in the landscape, and a selection ratio < one indicates that the forage was used proportionately less than its availability in the landscape. Selection for food grains consumed from the households was not assessed as they had a very low total feeding sign. As the total feeding sign of the orchid consumed was low (four) and it requires a unique quantification method for availability, the selection was not assessed for the orchid species as well. Forage selection was calculated both at a landscape level and separately in the different LULC types (forest, semi-open forest, open forest, tea estate, and village).

#### Ethics statement

We followed all national and international ethical guidelines during this research. Since the Asian elephant is a Schedule I species and certain parts of the research was conducted within the Protected Areas, according to the Wildlife (Protection) Act, 1972, required permission was obtained from the concerned Forest Department. The Forest Department’s permission letter number is 1694/140/OCB/2019 dated 11.11.2019 with memo number C-28011/09/2019 from Principal Chief Conservator of Forests (Wildlife) and Chief Wildlife Warden, Bikash Bhawan, Kolkata, West Bengal. The methodology followed to study the elephants has been approved by the research and ethics committee of SACON.

## Results

### Forage use

A total of 150.01 km of feeding trails were surveyed with 44.11 km in the forest, 27.78 km in the semi-open forest, 13.65 km in the open forest, 36.54 km in the tea estate, and 27.93 km in the village (Fig 1). The total number of trails walked in the forest, semi-open forest, open forest, tea estate, and village were 27, 30, 17, 15, and 29 respectively. The range of trail lengths in the forest was 0.14–3.60 km, the semi-open forest was 0.06–2.74 km, the open forest was 0.1–2.3 km, the tea estate was 0.19–6.3 km and the village was 0.04–5.5 km. The total feeding signs recorded were 3313 with 1543 in the forest, 365 in the semi-open forest, 63 in the open forest, 187 in the tea estate, and 1155 in the village.

**Fig 4:**
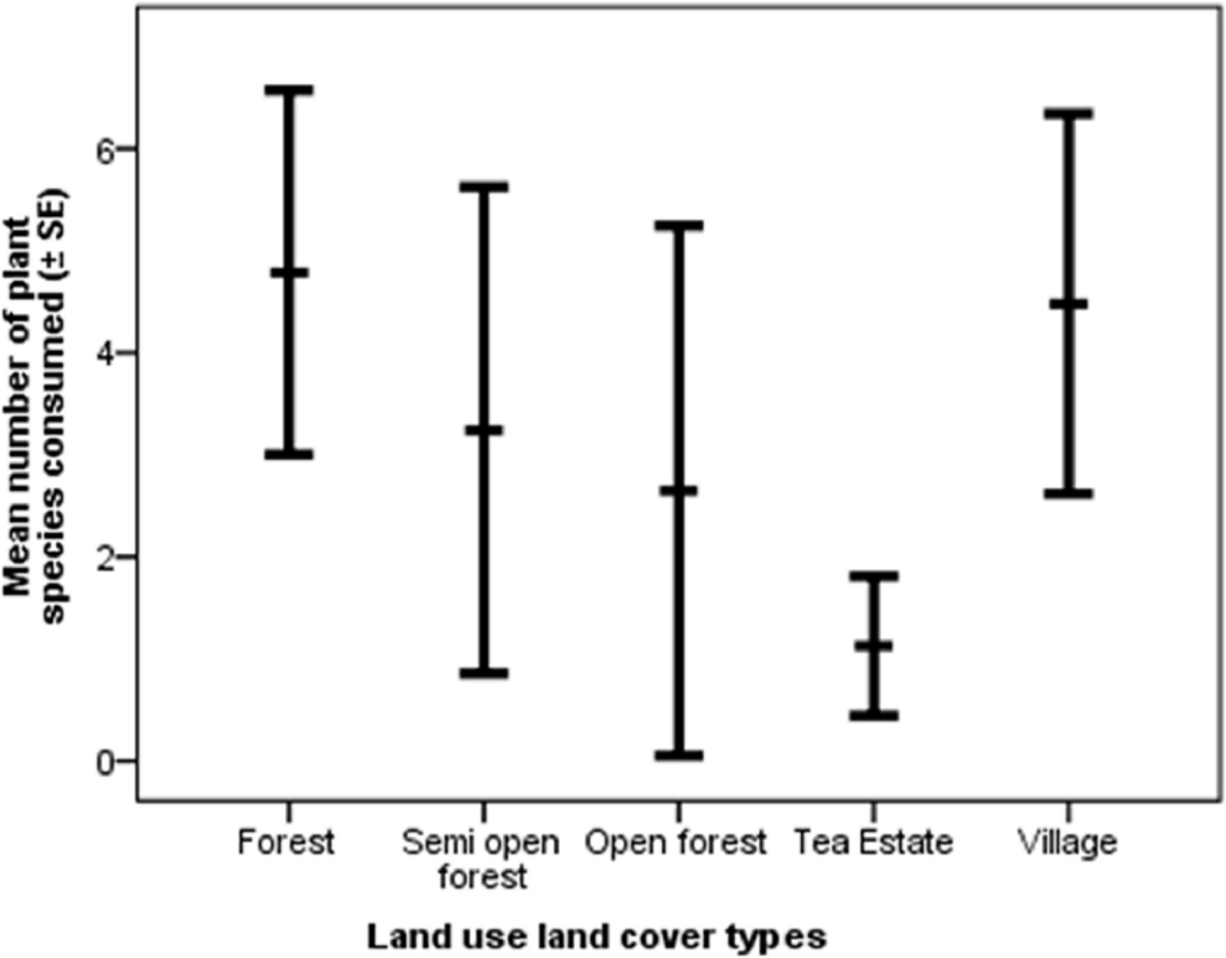
Mean number of plant species consumed per trail by Asian elephant.

**Fig 5:**
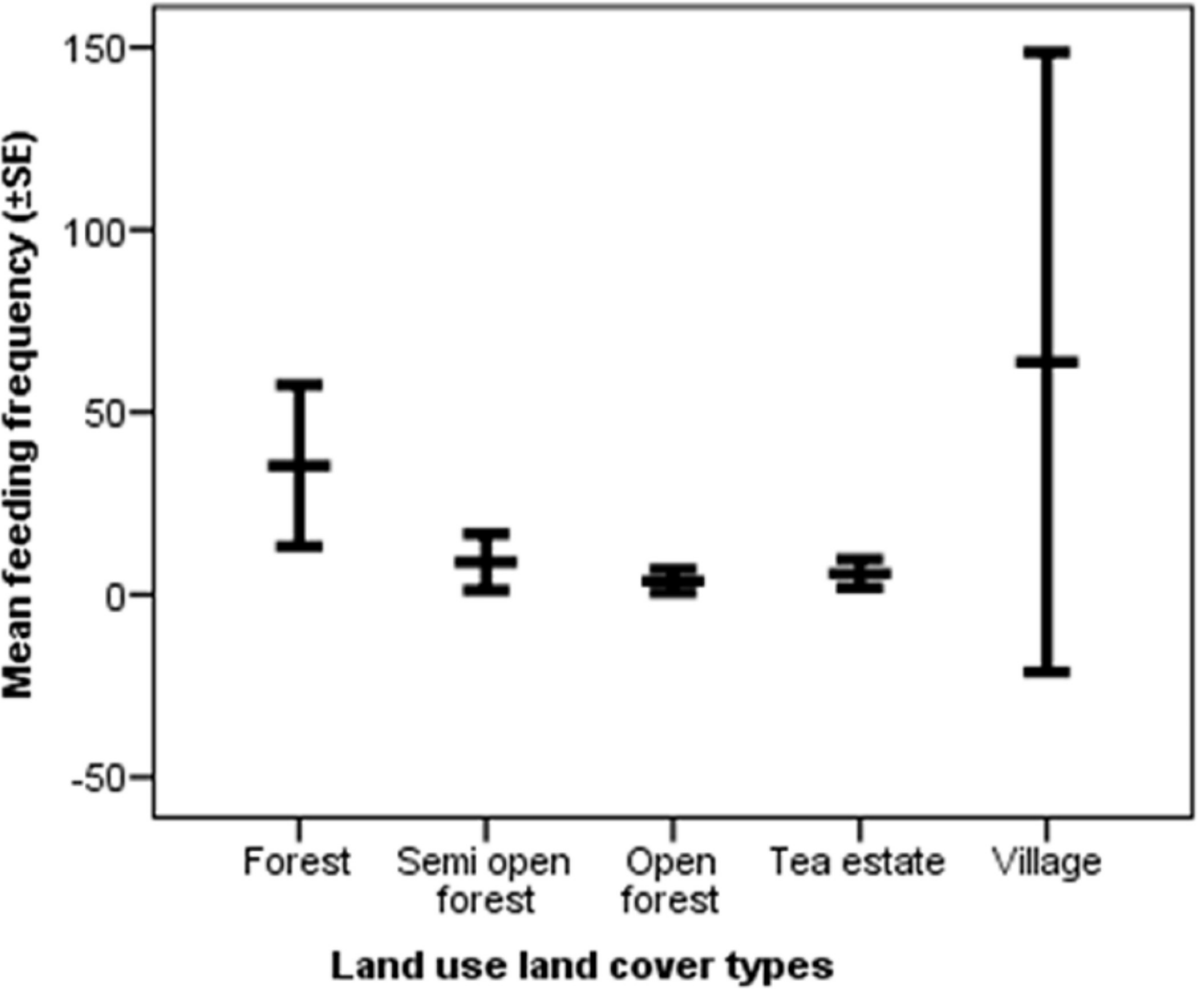
Mean feeding frequency of Asian elephant per trail in different land use and land cover types.

The total number of species recorded to be consumed by Asian elephants across different LULC types was 132 (including 3 varieties of food grain) (S2 Table), of which 21 plant species made up 85.3% of the total feeding signs recorded and 41 plant species were observed to be consumed only once. The total number of plant species consumed in the forest, semi-open forest, open forest, tea estate, and village were 86, 49, 19, 19, and 21 respectively. The mean number of the plant species consumed by an elephant in the forest, semi-open forest, open forest, tea estate, and village were 4.79±0.89_SE_, 3.24±1.19_SE_, 2.65±1.30_SE_, 1.13±0.34_SE_, and 4.48±0.93_SE_ respectively that significantly varied from each other (H = 22.26, df = 4, p<0.001) (Fig 4). The mean feeding frequency in the forest, semi-open forest, open forest, tea estate, and village were 40.51±9.42_SE_, 12.14±9.42_SE_, 3.31±1.44_SE_, 5.79±1.95_SE_, 50.15±22.85_SE_ respectively which significantly varied between the groups (H = 26.99, df = 4, p<0.001) (Fig 5). The percentage of trees, herbs, climbers, bamboos, palms, orchid and food grain consumed were 39.55%, 44.10%, 7.94%, 2.84%, 5.23%, 0.13%, 0.25% respectively.

The number of plant species for which fruit, leaf, branch, bark, stem, root, and flower was consumed was 6, 90, 51, 22, 13, 30, and 1 respectively (S2 Table). For 59 plant species, more than one plant part was consumed. Fruits were consumed for 6 species. Roots were consumed by completely uprooting the plants to access them. Among many species whose roots were consumed by the elephants, some, which were consumed at a sapling stage itself, were also of high timber value like *Shorea robusta*, *Tectona grandis*, *Lagerstroemia speciosa*, *Acacia catechu*. Some other species whose roots were consumed were *Ardisia solanacea*, *Acacia pennata*, *Macaranga denticulta*, *Mallotus philippensis*, and *Leea indica*. *Mimosa pudica* was the only species whose flower was consumed. Among the species found in private land, for *Musa sp*., fruit, the soft pith inside stem and leaves were consumed, for *Areca catechu* leaves and the soft pith inside stem was consumed and for the Bamboo species, mostly leaves were consumed.

The diet composed of 47.27% monocots and 52.73% dicots across different LULC types. In the forest, semi-open forest, open forest, tea estate and village, the percentage of dicot-monocot consumed were 28.83%-71.17%, 83.28%-16.71%, 79.36%-20.63%, 6.42%-93.58% and 81.17%-18.83% respectively.

Elephants consumed more browse (90.77%) than graze (9.23%) across different LULC types. In the forest, semi-open forest, open forest, tea estate and village, the percentage of browse-graze consumed were 84.83%-15.16%, 82.54%-17.46%, 93.15%-6.85%, 81.28%-18.72% and 100%-0% respectively.

The total survey effort of feeding trails for herds was 70.17 km and for male was 79.77 km. As the effort in different land use and land cover types was on the basis of opportunistic reports and not biased towards herd or male, it can be a proxy for land use and land cover that herds and males of the Asian elephant population used. There was no significant difference observed between use by male and herd in the forest (χ^2^ = 0.09, df = 1, p>0.05), semi-open forest (χ^2^ = 0.33, df = 1, p>0.05) and open forest (χ^2^ = 0.69, df = 1, p>0.05), whereas there was significant difference observed in tea estate (χ^2^ = 6.08, df = 1, p<0.05) and village (χ^2^ = 10.70, df = 1, p<0.05). Herds used tea estates more and males used villages more. The top five plant species consumed by the herd were *Phrynium pubinerve*, *Alpinia nigra*, *Dillenia indica*, *Areca catechu*, and *Isachne sp*. and the top five plant species consumed by males were *Acacia catechu*, *Phrynium pubinerve*, *Solanum tuberosum*, *Oplismenus burmanii*, and *Musa sp*.

### Plant species available

The total number of plots sampled was 123 of 0.1 ha (31 in forest, 24 in semi-open forest, 26 in open forest, 22 in tea estate, and 20 in village), which encompass an area of 12.3 ha (Fig 1).

**Fig 6:**
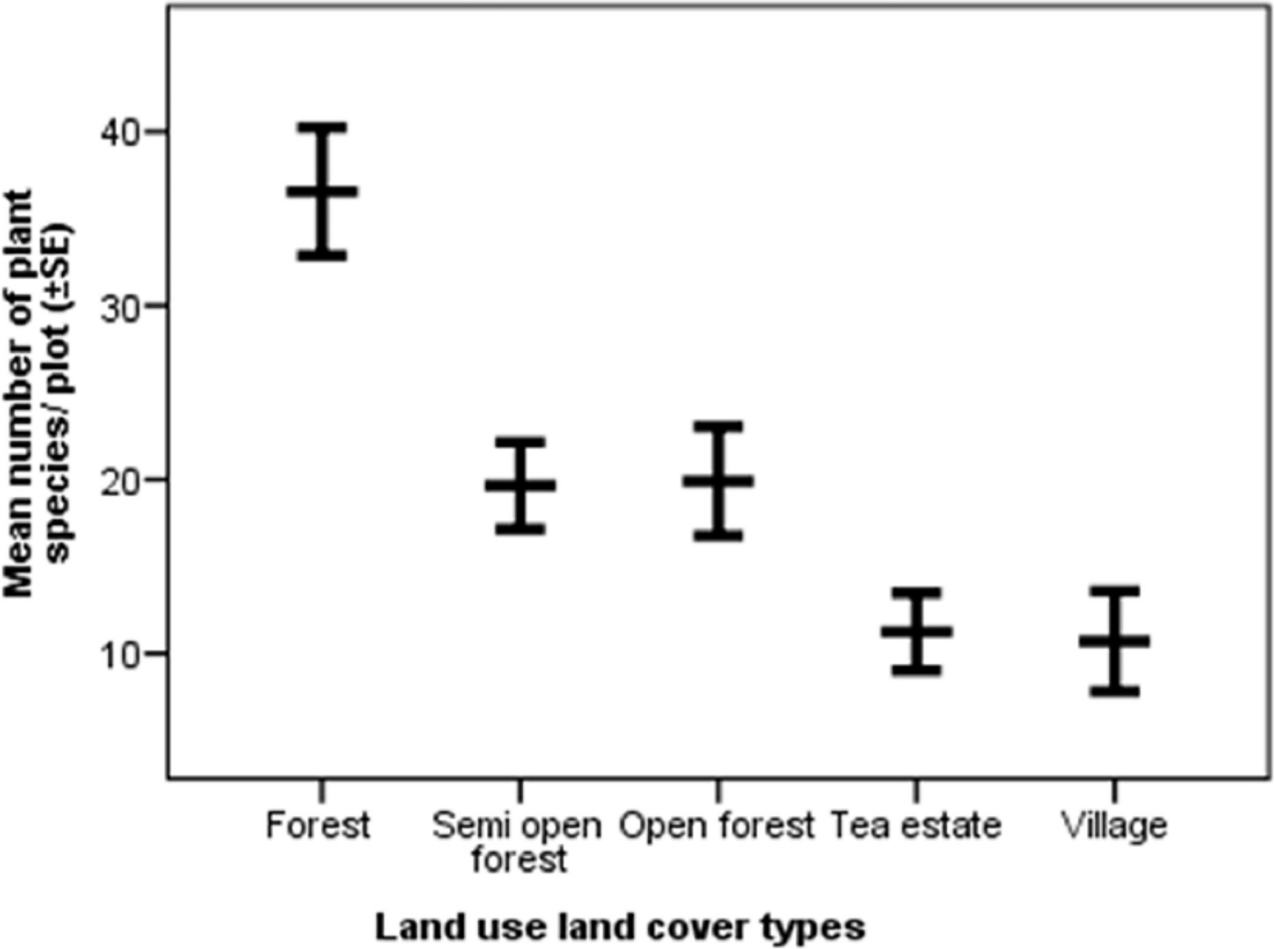
Mean (±SE) number of plant species/ plot recorded in different land.

A total of 286 plant species were recorded from the sampled area across different LULC types. The total number of plant species recorded in the forest, semi-open forest, open forest, tea estate, and village were 157, 133, 137, 76, and 80 respectively. The mean number of plant species recorded in the forest, semi-open forest, open forest, tea estate, and village per plot were 34.96±1.94_SE_, 20.83±1.41_SE_, 21.04±1.54_SE_, 11.25±1.11_SE_, and 10.70±1.44_SE_ respectively that significantly varied between groups (F = 44.13, df = 4, p< 0.001)(Fig 6). The average number of plant species present in the forest was the highest, followed by similar semi-open forests and open forest, tea estate, and village had a comparable number of species.

**Fig 7:**
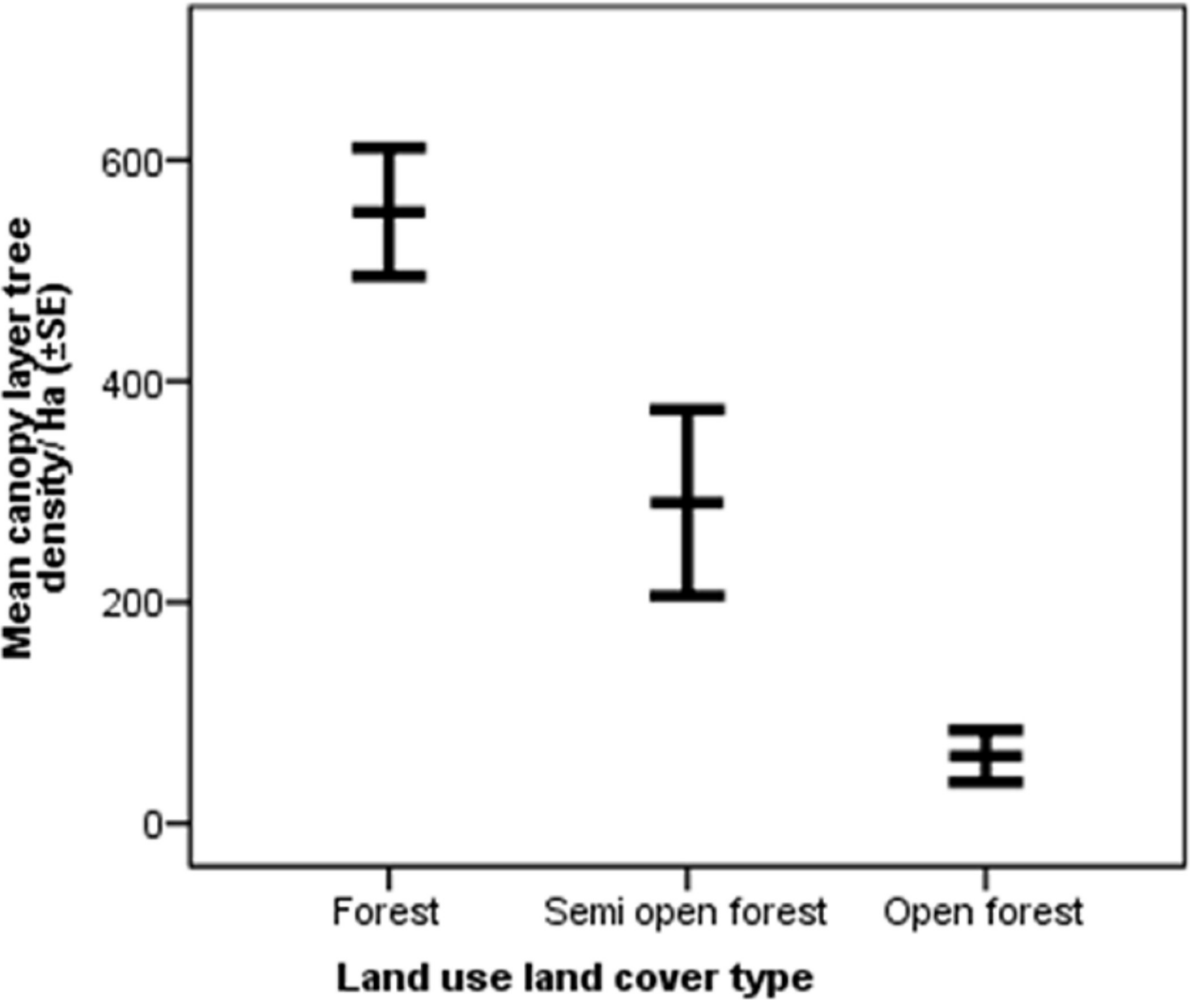
Mean (±SE) canopy layer tree density in the forest, semi-open forest, and open forest.

**Fig 8:**
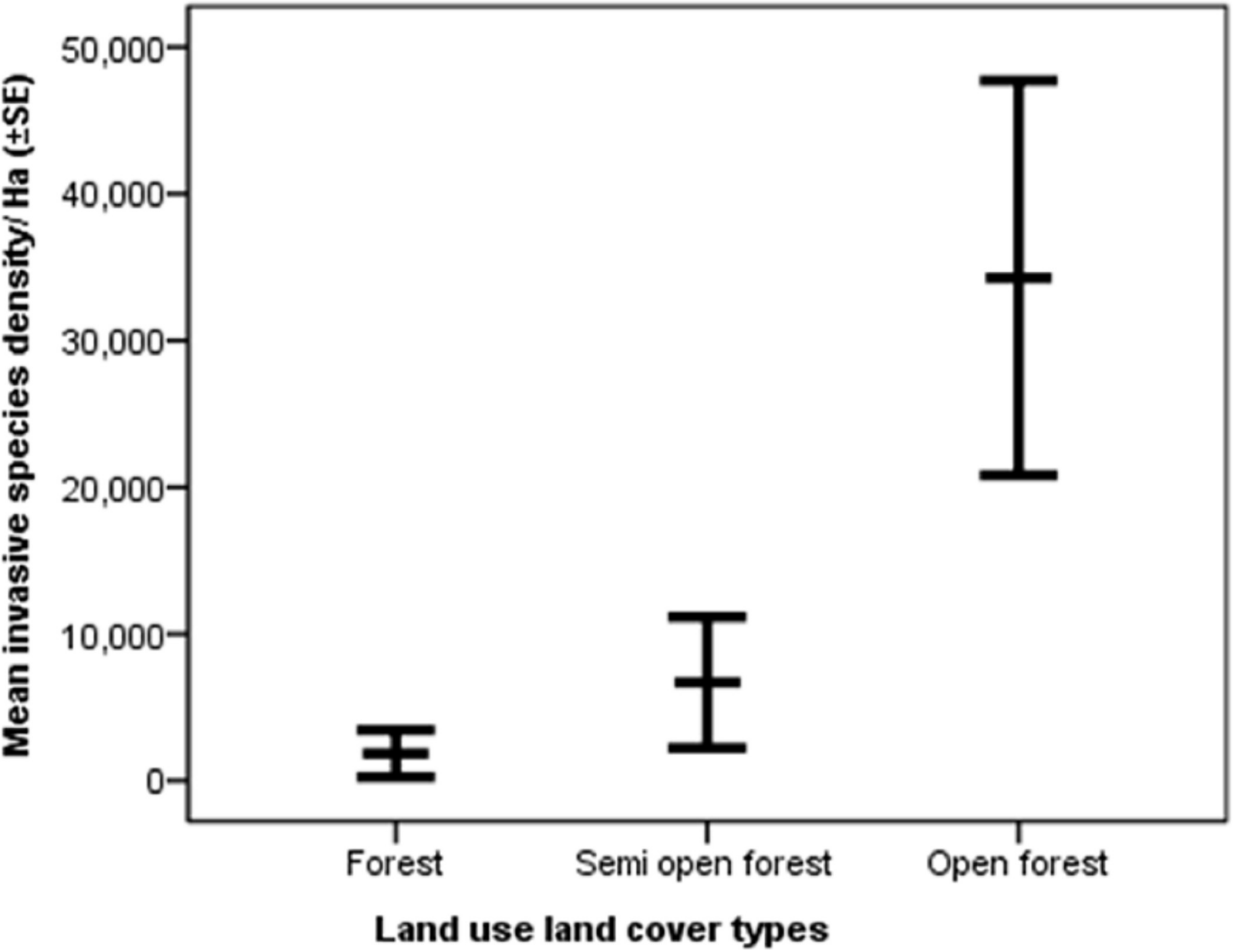
Mean (±SE) invasive species density in the forest, semi-open forest, and open forest.

The mean canopy tree density was highest in the forest (552.95±29.00_SE_) followed by semi-open forest (290.00±42.10_SE_) and open forest (60.83±11.75_SE_) (Fig 7). The mean invasive species density was highest in the open forest (34262.50±6721.86_SE_) followed by semi-open forest (6691.66±2234.31_SE_) and forest (1845.83±796.64_SE_) (Fig 8).

The percentage of available dicots-monocots recorded in the forest, semi-open forest, open forest, tea estate and village were 56.60%-43.39%, 76.41%-23.59%, 68.39%-31.61%, 82.63%-17.37% and 76.48%-23.51% respectively.

The percentage of available browse-graze recorded in forest, semi-open forest, open forest, tea estate and village were 67.15%-32.85%, 59.73%-40.27%, 32.71%-67.29%, 38.82%- 61.18% and 65.83%-34.17% respectively.

### Forage selection

Among the 96 plant species for which selection was assessed, 19 plant species had a selection ratio of less than one implying that they were used proportionately less than their availability in the environment. 77 species had a selection ratio of more than one implying that they were used proportionately more than their availability. 49 species had a selection ratio between one and 100, seven species had a selection ratio between 100 and 200, 16 species had a selection ratio between 200 and 1000, and five plant species had a selection ratio of more than 1000 (S2 Table). The top five species selected in different LULC types are provided in Table 1.

**Table 1:** Representing the species with the top five selection ratios in different land use and land cover type.

Land use and land cover types	Total no. of plant species consumed	No. of plant species- selection assessed	Selection ratio (>1 - <0)	Plant species with top 5 selection ratio
Forest	86	54	14–40	*Magnifera indica*, *Alstonia scholaris*, *Ficus virens*, *Bombax ceiba*, *Terminalia alata*
Semi open forest	49	30	4–26	Chiuri, *Bauhinia sp*., *Sterculia villosa*, *Syzigium cumini*, *Tectona grandis*
Open forest	19	12	12	*Ailanthus integrifolia*, *Callicarpa arborea*, *Bambusa bambos*, *Shorea robusta*, *Lagerstroemia paviflora*
Tea estate	19	16	16	*Musa sp*., *Bambusa nutans*, *Melocanna baccifera*, *Pandanus* furcatus, *Albizia procera*
Village	21	10	10	*Bambusa balcooa*, *Tectona grandis*, *Melocanna baccifera*, *Bambusa nutans* and *Areca catechu*

Out of the 28 plant species that had a selection ratio of more than 100, 14 species were available only in the forest land and nine plant species were available only in the private land and five species were available in both private and forest land. *Terminalia alata*, *Bambusa bambos*, *Ardisia solanacea*, *Phrynium pubinerve*, *Ficus elastica*, *Oroxylum indicum*, *Acacia pennata*, *Macaranga denticulate*, *Bauhinia sp*., *Chonemorpha fragrans*, *Dillenia indica*, *Smilax perfoliata*, *Walsura tabularis*, and *Dendrocalamus sp*. were available only in forest land whereas *Bambusa balcooa*, *Melocanna baccifera*, *Bambusa nutans*, *Pandanus furcatus*, *Musa sp*., *Areca catechu*, *Artocarpus heterophyllus*, *Solanum lycopersicum*, *Solanum melongena* were available only in private land. *Ficus virens*, *Tectona grandis*, *Mangifera indica*, *Acacia catechu*, and *Emblica officinalis* were available in both private and forest land.

## Discussion

### Forage use

The present study recorded 132 plant species (including three species of food grains) to be consumed by Asian elephants in the dry season. The recorded number of species consumed by Asian elephants highly varies between study sites e.g., 57 in Nepal [27], 26 in Vietnam [21], 71 in central India [24], 106 in China [20], 112 in southern India [19], and 182 in Borneo [26]. As elephants are monogastric hindgut fermenters, they are poor at dealing with defensive toxins produced by plants which can be resolved by increasing food diversity thereby reducing the intake of particular toxins [30, 45]. Although the diet is highly diverse across its range, the difference in the number of species is reflective of the diversity and composition (nutrients and secondary compounds) of the plants available in the particular landscape they inhabit [30] and also due to the differences in the study methodology [27].

Twenty-one plant species contributed 85.3% of the total feeding signs recorded while 41 plant species were recorded to be consumed just once. Elephants are known to feed on the plants most familiar to them while continuously sampling other plants [2]. In particular, the Asian elephant is known to consume more than 100 plant species but only a few species constitute the majority of their forage intake [15]. For example, 85% of their diet is composed of 25 species in south India [19] and 15 species in northern West Bengal [22].

We found that 39.55% of the plant species consumed in the study area constituted of trees, while the rest was composed of bamboos, palms, herbs, orchids, and food grains. Tree species have been found to dominate the forage intake in tropical dry forests [24] while non-tree species dominated the diet in tropical moist forests [26, 46]. Our observation of non-tree species dominating the forage intake could be because of two reasons: i) the forest fragments are of moist tropical type, and ii). the previous studies recorded forage use only in forest land but we recorded in a multi-use landscape. In private land, non-tree species like *Musa sp*., *Areca catechu*, and different species of bamboo dominated the diet.

Asian elephants are sometimes selective of the plant part they consume [43], with less nutritious diets, rich in indigestible fiber predominant in the dry season [47]. Elephants are known to feed on fresh foliage more while browsing and consume dry branches and twigs when the need arises in the dry season [2]. This is consistent with our findings, where we recorded elephants to feeding on the leaves of most of the plant species followed by branch, root, bark, and stem. *Mimosa pudica* was the only species whose flower was consumed similar to previous studies [19]. The total feeding sign recorded for *Dillenia indica* is consistent with the findings from Buxa Tiger Reserve (lies on the eastern side of the study area), where elephants were observed to remove 63.3% *Dillenia indica* fruits [48]. The lack of fruits in the diet could be because of the unavailability of fruiting trees and because Asian elephants are known to be less frugivorous in contrast to the African elephant [49]. Although elephants are known to consume roots [2], this is the first study to extensively document root consumption by Asian elephants from a large number of plant species. This indicates that elephants have a high impact on the vegetation composition of the forests in the landscape.

Overall, the plant species consumed by elephants are composed of 47.27% monocots and 52.73% dicots. Elephants are known to prefer monocots for their relatively high carbohydrate content [50] and in moist tropical forests Asian elephants have been found to have a strong impact on monocots diversity [25, 26]. This study found in the forest, elephants consumed more monocots whereas, in semi-open forests and open forests, they consumed more dicots although the availability of monocots was lesser than dicots in all these LULC types. The difference could probably be because of the lesser abundance of the palatable monocots in semi-open forests and open forests.

During the present study, overall plants species consumed by elephants constituted 90.77% browse and 9.23% graze. The intake of browse was more in all LULC types. In the forest, semi-open forest, and village, more browse was available whereas, in open forest and tea estate, more graze was available. In open forest and tea estates, graze species were probably not consumed in proportion to their availability as the species were not palatable. Browse is known to dominate the diet in the dry season [19, 47] and our finding is consistent with the findings from Buxa Tiger Reserve where 93% of the dry season intake was found to be browse [22].

The sexes in Asian elephants have been long predicted to have different nutritional requirements and habitat use patterns which, could be due to the greater need of females and young to avoid predators and anthropogenic disturbances and high nutritional requirement of lactating females [2]. We found males used villages significantly more, whereas herds used tea-estates significantly more. Males came to villages particularly for foraging, while herds foraged opportunistically in tea estates while moving between adjacent forest fragments. As a result, four of the five most commonly consumed plant species differed between males and herds. In a fragmented landscape, herds have been found to occur significantly higher in medium-forage, medium-disturbance areas [51] and male elephants are known to adopt a high-risk, high-gain foraging strategy [52]. During the dry season, most of the croplands in the landscape remain uncultivated except for some households growing mustard and vegetables for household consumption. *Musa sp*. is not grown commercially in plantations in the landscape but most houses have their own plants. Similarly, *Areca catechu* is planted within house compounds except a few households who grows them at a small scale for commercial purpose. At the initiation of the dry season, paddy is harvested in the landscape and stored in houses. Food grains consumed from households was just 0.25%. However, elephants were found to frequent private land to feed on *Musa sp*., *Areca catechu*, different species of bamboo among others which could lead to accidental encounters with humans and subsequent negative interactions. Hence, interventions must be made to ensure that these plants are not planted too close to houses and methods should be developed to protect them. However, the scenario is expected to be completely different when maize and paddy are cultivated in the landscape leading to sufficient negative interaction, especially in the form of economic loss due to crop depredation.

### Forage selection

The logistical problem in assessing the distribution and availability of plant species has long prevented the quantification of forage selection [2], and this is one of the few studies to examine forage selection by the Asian elephant. Of the 29 species that had a selection ratio > 100, 50% were available only in forest land, 32.14% were available only in private land and the rest were available in both. Thus, in terms of forage availability, non-forested areas play only a subsidiary role in supporting the foraging of elephants and are not substitutes for forest land. Conversely, the presence of 32.14% of the selected forage species in private land alone demands the development of measures and strategies to guard them or ensure the cultivation of these species at a distance from houses so that accidental encounters between people and elephants could be avoided when elephants venture close to settlements to access them.

The present study shows that among the plants for which selection was analysed, 80.21% of species were consumed more than their availability in the landscape. The results indicate selectivity in the diet of the Asian elephants during the dry season in the landscape. Elephants like other large generalist herbivores are thought to feed on plants that are usually available in abundance [2, 6]. Although our finding indicates contradiction to this knowledge, it can only be established over a repeated multi-season study.

### Differences across land use and land cover types

On average, elephants consumed plant species more frequently in villages followed by forests, semi-open forests, tea estates, and open forests. However, the high variance in average feeding frequency in the village could be because elephants mostly used villages as passage and foraged opportunistically sometimes. The average number of plant species consumed was highest in the forest followed by the village, semi-open forest, open forest, and tea estate. Although the average number of species consumed in the forest (4.79±0.89SE) and village (4.48±0.93SE) was similar, the total number of species consumed in the forest (86) was much higher than that in the village (21). In terms of availability, forest supported the most number of plant species followed by semi-open forest, open forest, village, and then tea estate. The canopy layer tree density reduced from forest to semi-open forest and open forest, while the invasive species density increases from forest to semi-open forest and open forest. Invasive species are known to cause habitat modification and native plant species loss [53] and the findings of this study indicate a similar mechanism occurring in semi-open forests and open forests. The requirement of high diversity of food plants to ensure access to different nutrients for elephants [2], makes it important to adopt appropriate habitat management measures in semi-open forests and open forests to curb growth in invasive species density and restore them to ensure forage availability for elephants.

## CAVEAT

The study was conducted during the dry season and the inferences should be restricted for the same period alone as forage use by elephants is known to differ across seasons based on the availability and nutritional value of the plants [2, 11]. However, this does not dilute the management implication suggested. Direct behavioural observation and diet analysis could have complemented the present study. However, direct behavioural observation was not possible due to the closed canopy system of the forests in the study area and diet analysis was beyond the scope of the study.

## Conclusion

This was the first study that assessed forage selection by Asian elephants in a multi-use landscape. The importance of non-tree species in the moist tropical forest was reinforced. High selectivity for forage species was found contradictory to the knowledge that elephants are generalist feeders. Non-forested areas played only a subsidiary role and not a substitute for forest land in terms of supporting the availability of the selected forage species. However, as some selected forage species were available only in the private land, it demands the development of strategies to prevent negative interactions in terms of economic loss or accidental attacks when elephants come to forage on these species. In forest land, appropriate habitat management measures are required in semi-open forest and open forests to ensure forage availability for elephants. Future research on Asian elephants in this landscape must be focussed on the following: i) assessment of forage selection in the wet season as the scope of this study was restricted to the dry season, ii) role in seed dispersal and forest regeneration and iii) nutritional composition of the foraged species to understand if the elephant population is undergoing nutritional stress. Such information would not just expand our knowledge about the ecosystem services provided by Asian elephants which is limited [48] but help in developing better conservation interventions as well.

## Supporting information

S1 Table Data of the 30 training site locations in the 5 land use land cover types. (XLSX)

S2 Table Data of plants species consumed by Asian elephant along with plant part consumed, number of feeding signs in different land use and land cover types (F- Forest, SOF–Semi-open Forest, OF–Open Forest, TE–TE, V- Village), total feeding signs (Total FF) and selection ratio. (DOCX)

S1 Text Forage availability data extrapolation calculation. (DOCX)
