# Supplementary material for: Trunk picking from a truncating menu: Dry season forage selection by Asian elephant in a multi-use landscape
Source: PLoS One. 2022 Jul 8;17(7):e0271052. doi: 10.1371/journal.pone.0271052 (PMC9269951; doi:10.1371/journal.pone.0271052)
Supplement: S2 Table — (DOCX) [file pone.0271052.s002.docx]

Plants species consumed by Asian Elephant along with plant parts consumed, number of feeding signs in different land use and land cover types (F- Forest, SOF – Semi-open Forest, OF – Open Forest, TE – TE, V- Village), total feeding signs (Total FF) and selection ratio.

| SL No | Local Name | Scientific Name | Plant part consumed | F | OF | SOF | TE | V | Total FF | Selection Ratio |
| --- | --- | --- | --- | --- | --- | --- | --- | --- | --- | --- |
| 1 | Paka Sash | *Terminalia alata* | Leaf, Root | 1 |  | 1 |  |  | 2 | 4070150 |
| 2 | Tel baas | *Bambusa balcooa* | Leaf | 1 |  |  | 14 | 17 | 32 | 39408.42 |
| 3 | Filling baas | *Melocanna baccifera* | Leaf | 6 |  |  | 1 | 5 | 12 | 4926.06 |
| 4 | Kata baas | *Bambusa bambos* | Leaf | 1 | 2 | 20 |  | 1 | 24 | 2955.64 |
| 5 | Makla baas | *Bambusa nutans* | Leaf |  |  |  | 12 | 10 | 22 | 1354.67 |
| 6 |  | *Pandanus furcatus* | Leaf |  |  |  | 3 |  | 3 | 923.64 |
| 7 | Haringudi | *Ardisia solanacea* | Branch, Leaf, Root | 7 |  |  |  |  | 7 | 862.06 |
| 8 | Banana | *Musa sp.* | Fruit, Leaf, Stem |  |  |  | 31 | 64 | 95 | 801.33 |
| 9 | Mechia Pata | *Phrynium pubinerve* | Leaf, Stem | 541 |  |  |  |  | 541 | 774.71 |
| 10 | Supari | *Areca catechu* | Leaf, Stem |  |  |  | 52 | 117 | 169 | 754.08 |
| 11 | Kabra | *Ficus virens* | Bark, Leaf | 1 |  |  |  |  | 1 | 615.76 |
| 12 | Labar | *Ficus elastica* | Branch | 1 |  |  |  |  | 1 | 615.76 |
| 13 | Totola | *Oroxylum indicum* | Leaf |  |  | 2 |  |  | 2 | 615.76 |
| 14 | Arare Kara | *Acacia pennata* | Bark, Branch, Leaf, Root | 4 | 3 | 12 |  |  | 19 | 467.98 |
| 15 | Teak | *Tectona grandis* | Bark, Branch, Leaf, Root, Stem | 2 | 1 | 61 |  | 11 | 75 | 441.94 |
| 16 | Moleto | *Macaranga denticulata* | Bark, Branch, Leaf, Root, Stem | 33 |  | 11 |  |  | 44 | 320.64 |
| 17 | Taki | *Bauhinia sp.* | Branch, Leaf | 6 |  | 2 |  |  | 8 | 289.77 |
| 18 | Kathal | *Artocarpus heterophyllus* | Leaf |  |  |  |  | 1 | 1 | 246.31 |
| 19 | Dudhe Lahara | *Chonemorpha fragrans* | Branch, Leaf | 64 |  | 2 |  |  | 66 | 239.06 |
| 20 | Aam | *Mangifera indica* | Branch, Leaf | 1 |  |  |  |  | 1 | 205.26 |
| 21 | Chalta | *Dillenia indica* | Fruit, Leaf | 113 | 15 | 3 |  |  | 131 | 201.16 |
| 22 | Khair | *Acacia catechu* | Root |  |  | 4 |  | 630 | 634 | 161.39 |
| 23 | Amra/ Amloki | *Emblica officinalis* | Branch, Leaf | 1 |  |  |  |  | 1 | 123.16 |
| 24 | Baghe kata | *Smilax perfoliata* | Leaves | 1 |  |  |  |  | 1 | 123.16 |
| 25 | Tomato | *Solanum lycopersicum* | Fruit |  |  |  |  | 20 | 20 | 123.16 |
| 26 | Falame | *Walsura tabularis* | Branch, Leaf | 1 |  |  |  |  | 1 | 123.16 |
| 27 | Jongli baas | *Dendrocalamus sp.* | Leaf | 1 |  |  |  |  | 1 | 123.16 |
| 28 | Brinjal | *Solanum melongena* | Fruit |  |  |  |  | 49 | 49 | 100.58 |
| 29 | Chatim | *Alstonia scholaris* | Leaf | 3 |  |  |  |  | 3 | 80.32 |
| 30 | Gualo | *Callicarpa arborea* | Branch, Leaf |  | 1 | 2 |  |  | 3 | 78.61 |
| 31 | Simul | *Bombax ceiba* | Bark, Leaf | 1 |  | 1 |  | 1 | 3 | 76.97 |
| 32 | Sindure | *Mallotus philippensis* | Bark, Branch, Leaf, Root, Stem | 19 | 2 | 36 |  |  | 57 | 76.14 |
| 33 | Gomari | *Gmelina arborea* | Bark, Branch |  |  | 1 |  |  | 1 | 68.42 |
| 34 | Purundi | *Alpinia nigra* | Leaf, Stem | 180 |  | 3 |  |  | 183 | 66.09 |
| 35 | Bon Kathal/ Barhar | *Artocarpus lakoocha* | Leaf, Branch |  |  |  |  | 1 | 1 | 61.58 |
| 36 | Ful jharu/ kucho | *Thysanolaena latifolia* | Leaf |  | 1 |  |  |  | 1 | 41.06 |
| 37 | Patpate Sirish | *Albizia lucidior* | Branch, Leaf | 1 |  | 72 | 1 |  | 74 | 36.43 |
| 38 | Goleni/ Hatubhanga | *Leea indica* | Bark, Branch, Leaf, Root | 42 |  | 14 |  |  | 56 | 34.84 |
| 39 | Ful ghas | *Holmskioldia sanguinea* | Leaf | 1 |  | 1 |  |  | 2 | 30.79 |
| 40 | Charpate | *Carex sp.* | Leaf | 32 |  |  |  |  | 32 | 24.48 |
| 41 | Bakshi Kata | *Caesalpinia cucullata* | Bark, Branch | 1 |  | 3 |  |  | 4 | 23.46 |
| 42 | Ram Suntala | *Chisocheton cumingianus* | Root | 1 |  |  |  |  | 1 | 22.4 |
| 43 | Jarul | *Lagerstroemia speciosa* | Bark, Branch, Leaf, Root | 8 | 3 | 27 | 1 |  | 39 | 19.53 |
| 44 | Gokul | *Ailanthus integrifolia* | Stem |  | 1 |  |  |  | 1 | 19.25 |
| 45 | Bash pate ghas | *Molineria capitulata* | Leaf | 3 |  |  |  |  | 3 | 16.8 |
| 46 | Tatari | *Dillenia pentagyna* | Bark, Branch, Root | 4 |  |  |  |  | 4 | 16.1 |
| 47 | Thali | *Turpinia pomifera* | Leaf | 1 |  |  |  |  | 1 | 15.4 |
| 48 | Haldikath | *Morinda augustifolia* | Bark, Branch, Leaf | 13 |  | 1 |  |  | 14 | 14.74 |
| 49 | Sida/ Burdamero | *Lagerstroemia parviflora* | Branch |  | 2 |  |  |  | 2 | 14.24 |
| 50 | Kotush/ Musure | *Castanopsis hystrix* | Bark, Branch, Leaf, Root | 4 |  | 1 |  |  | 5 | 12.39 |
| 51 | Maidulu Kara/Myna Kara | *Meyna spinosa* | Branch, Leaf, Root | 1 | 2 | 1 |  |  | 4 | 11.2 |
| 52 | Jhingeni | *Eurya acuminata* | Branch | 1 |  |  |  |  | 1 | 10.18 |
| 53 | Sada Sirish | *Albizia procera* | Bark, Leaf, Root |  | 1 |  | 2 |  | 3 | 8.31 |
| 54 | Sal | *Shorea robusta* | Bark, Branch, Root | 14 | 1 | 24 |  |  | 39 | 7.66 |
| 55 | Kalo Sirish | *Albizia lebbeck* | Leaf |  |  |  | 1 |  | 1 | 6.13 |
| 56 | Kumbi | *Careya arborea* | Branch |  |  | 1 |  |  | 1 | 4.14 |
| 57 | Hare Khirra | *Holarrhena pubescens* | Leaf |  |  | 1 |  |  | 1 | 3.93 |
| 58 | Gayo | *Bridelia retusa* | Bark, Branch, Leaf | 5 | 2 | 3 |  |  | 10 | 3.57 |
| 59 | Jamun | *Syzygium cumini* | Branch, Leaf, Root | 5 |  | 1 |  |  | 6 | 3.39 |
| 60 | Kaulo | *Persea gamblei* | Branch, Leaf | 3 |  |  |  |  | 3 | 2.93 |
| 61 | Khakar | *Albizia odoratissima* | Leaf |  |  |  | 1 |  | 1 | 2.89 |
| 62 | Lali | *Aglaia spectabilis* | Branch, Leaf |  | 1 |  |  |  | 1 | 2.72 |
| 63 | Odal | *Sterculia villosa* | Bark, Branch | 1 | 1 | 1 |  |  | 3 | 2.71 |
| 64 | Bashe Bonsho | *Isachne sp.* | Leaf, Stem | 96 |  | 4 |  |  | 100 | 2.53 |
| 65 | Kaphol | *Myrica sp.* | Fruit | 1 |  |  |  |  | 1 | 2.52 |
| 66 | Choulane | *Litsea lancifolia* | Branch, Root | 14 |  | 1 |  |  | 15 | 2.39 |
| 67 | Amkoili | *Cryptocarya sp.* | Branch, Leaf, Root, Stem | 2 |  |  |  |  | 2 | 2.34 |
| 68 | Archal/ Bandar khaja | *Antidesma acidum* | Root | 1 |  |  |  |  | 1 | 2.24 |
| 69 | Mikania/Asami lata | *Mikania micrantha* | Leaf, Stem | 17 |  |  |  |  | 17 | 2.22 |
| 70 | Jhakri kath | *Ocotea lancifolia* | Root |  |  | 1 |  |  | 1 | 1.49 |
| 71 | Pani Lahara | *Cissus repanda* | Branch, Leaf | 3 |  |  |  |  | 3 | 0.96 |
| 72 | Kutmero | *Litsea monopetala* | Branch, Leaf, Root |  | 2 |  |  |  | 2 | 0.93 |
| 73 | Lapche | *Polyalthia simiarum* | Bark |  |  | 1 |  |  | 1 | 0.86 |
| 74 | Bonsho ghas | *Oplismenus burmanii* | Leaf | 116 |  |  | 35 |  | 151 | 0.75 |
| 75 | Dhotisuro | *Setaria palmifolia* | Leaf | 1 |  |  |  |  | 1 | 0.69 |
| 76 | Lata Sirish | *Dalbergia stipulacea* | Branch, Leaf | 3 |  |  |  |  | 3 | 0.45 |
| 77 | Charchare Lahara | *Tetrastigma serrulatum* | Branch, Leaf | 4 |  | 1 |  |  | 5 | 0.32 |
| 78 | Jongli Bhatne | *Clerodendrum bracteatum* | Root | 3 |  |  |  |  | 3 | 0.16 |
| 79 | Ghatu pata | *Clerodendrum infortunatum* | Root | 7 |  |  |  |  | 7 | 0.15 |
| 80 | Gaujo | *Millettia extensa* | Leaf |  |  | 1 |  |  | 1 | 0.11 |
| 81 | Dheki Shak | *Diplazium esculentum* | Root, Stem | 3 |  |  |  |  | 3 | 0.06 |
| 82 | Jongli Pan | *Piper beteloides* | Leaf | 4 |  |  |  |  | 4 | 0.05 |
| 83 | Chepti Ghas | *Axonopus compressus* | Leaf |  |  | 12 |  |  | 12 | 0.04 |
| 84 | Lajjabati | *Mimosa pudica* | Flower | 1 |  |  |  |  | 1 | 0.04 |
| 85 | Kuro ghas | *Urena lobata* | Leaf | 1 |  |  |  |  | 1 | 0.02 |
| 86 | Cabbage | *Brassica oleracea* | Fruit |  |  |  |  | 3 | 3 | - |
| 87 | Coconut | *Cocos nucifera* | Branch |  |  |  |  | 1 | 1 | - |
| 88 | Dal (masoor) | *Lens culinaris* | Food grain |  |  |  |  |  |  | - |
| 89 | Deu Purundi | *Alpinia malaccensis* | Leaf | 1 |  | 1 |  |  | 2 | - |
| 90 | Flour (ata) | *Triticum aestivum* | Food grain |  |  |  | 1 |  | 1 | - |
| 91 | Guatemalan | *Tripsacum laxum* | Leaf | 50 |  |  |  |  | 50 | - |
| 92 | Jongli Dhan | *Coix sp.* | Leaf | 20 |  |  |  |  | 20 | - |
| 93 | Kukur diane | *Smilax macrophyla* | Leaf, Branch |  |  | 1 |  |  | 1 | - |
| 94 | Nima Bash | *Bambusa sp.* | Leaf |  |  |  |  | 1 | 1 | - |
| 95 | Peepal | *Ficus religiosa* | Bark, Branch |  |  |  |  | 2 | 2 | - |
| 96 | Potato | *Solanum tuberosum* | Root |  |  |  | 6 | 203 | 209 | - |
| 97 | Rai shak | *Brassica sp.* | Leaf |  |  |  | 15 |  | 15 | - |
| 98 | Rice | *Oryza sativa* | Food grain |  |  |  | 2 | 4 | 6 | - |
| 99 | Simul kandha | *Manihot esculanta* | Leaf, Root |  |  |  | 10 | 9 | 19 | - |
| 100 |  | *Asplenium sp.* | Leaf |  |  | 2 |  |  | 2 | - |
| 101 |  | *Pericampylus glaucus* | Leaf | 3 |  |  |  |  | 3 | - |
| 102 | Chana ghas/ Kasai |  | Branch, Leaf | 1 | 11 | 1 |  |  | 13 | 34.36 |
| 103 | Ghora ghas |  | Leaf | 10 |  |  |  |  | 10 | 30.79 |
| 104 | Jongli anaros |  | Leaf | 5 |  | 4 |  |  | 9 | 27.71 |
| 105 | Thakouli |  | Leaf, Branch | 3 |  | 1 |  |  | 4 | 2.8 |
| 106 | Birali Lahara |  | Leaf |  |  | 1 |  |  | 1 | 2.57 |
| 107 | Tite Lahara |  | Leaf, Branch | 1 |  | 1 |  |  | 2 | 2.06 |
| 108 | Jongli lichi |  | Branch, Leaf, Root | 7 |  |  |  |  | 7 | 1.7 |
| 109 | Kali Lahara |  | Branch, Leaf | 6 | 3 |  |  |  | 9 | 0.81 |
| 110 | Shiru ghas |  | Root |  |  | 10 |  |  | 10 | 0.38 |
| 111 | Kane ghas |  | Leaf, Stem | 4 | 10 |  |  |  | 14 | 0.22 |
| 112 | Akle ghas |  | Leaf |  |  | 3 |  |  | 3 | 0.08 |
| 113 | Aran/ Thesh |  | Branch | 1 |  |  |  |  | 1 | - |
| 114 | Basak Pata |  | Leaf | 1 |  |  |  |  | 1 | - |
| 115 | Burjo Lahara |  | Branch | 1 |  |  |  |  | 1 | - |
| 116 | Chiuri |  | Leaf |  |  | 4 |  |  | 4 | - |
| 117 | Dhakia Lorong |  | Leaf | 1 |  |  |  |  | 1 | - |
| 118 | Harkata |  | Leaf | 3 |  |  |  |  | 3 | - |
| 119 | Huku Lali |  | Branch | 1 |  |  |  |  | 1 | - |
| 120 | Jhirpitia |  | Leaf | 2 |  |  |  |  | 2 | - |
| 121 | Jiga |  | Bark, Root | 1 |  |  |  |  | 1 | - |
| 122 | Jongli bokshu |  | Leaf | 1 |  |  |  |  | 1 | - |
| 123 | Jongli Cha Pata |  | Branch | 1 |  |  |  |  | 1 | - |
| 124 | Jongli Sirish |  | Leaf, Root | 3 |  |  |  |  | 3 | - |
| 125 | Kata sule |  | Leaf | 1 |  |  |  |  | 1 | - |
| 126 | Mondola |  | Leaf | 1 |  |  |  |  | 1 | - |
| 127 | Nebara |  | Fruit |  |  |  |  | 1 | 1 | - |
| 128 | Unidentified Orchid |  | Leaf | 4 |  |  |  |  | 4 | - |
| 129 | Pitwai |  | Branch | 2 |  |  |  |  | 2 | - |
| 130 | Shit Lata |  | Leaf, Branch |  |  | 1 |  |  | 1 | - |
| 131 | Unidentified Bamboo |  | Leaf |  |  |  | 2 |  | 2 | - |
| 132 | Unidentified climber |  | Leaf | 1 |  |  |  |  | 1 | - |
