# Supplementary material for: Trunk picking from a truncating menu: Dry season forage selection by Asian elephant in a multi-use landscape
Source: PLoS One. 2022 Jul 8;17(7):e0271052. doi: 10.1371/journal.pone.0271052 (PMC9269951; doi:10.1371/journal.pone.0271052)
Supplement: S1 Text — (DOCX) [file pone.0271052.s003.docx]

Forage availability data extrapolation calculation

The abundance of all the plant species was assessed for the entire sampled area. The total number of plots sampled were 123 (31 in forest, 24 in semi-open forest, 26 in open forest, 22 in tea estate and 20 in village), which encompass an area of 12.3 ha. The abundance of the plant species found in the canopy, understory and ground layer was calculated in the following ways:

Canopy species:

Sum of all the records for a particular species in each plot

Understory species:

Total area sampled for understory species in each plot

= 25 m² x 4 = 100 m² = 0.01 ha

Total area sampled for understory species in the entire study area

= 123 x 0.01 ha = 1.23 ha

Therefore, total abundance of the understory species recorded

= ((recorded number of species) x 12.3)/1.23

= recorded number of species x 10

Ground species:

Total area sampled for ground species in each plot

= 1 m² x 5 = 5 m² = 0.0005 ha

Total area sampled for understory species in the entire study area

= 123 x 0.0005 ha = 0.0615 ha

Therefore, Total abundance of the understory species recorded

= ((recorded number of species) x 12.3)/ 0.0615

= recorded number of species x 200

If a plant species was present in two different layer (example: Shorea robusta as a fully grown tree in canopy layer and as a sapling in understory layer), then their total abundance in both the layer were summed up.
